# Experiential and Medical Knowledge of Shared Decision‐Making: Exploring Clinician and Service User Perspectives in NHS Community Mental Health Services

**DOI:** 10.1111/hex.70828

**Published:** 2026-08-31

**Authors:** Karishma Jivraj, Iris Gault, Mary Chambers

**Affiliations:** ^1^ King's College London; ^2^ Kingston University

## Abstract

**Background:**

A fundamental barrier to effective recovery outcomes for psychosis, is the lack of involvement of service users (SUs) in decisions made about their care. Despite policy recommendations, implementation of shared decision‐making (SDM) is inconsistent, under‐reported in mental health practice and thus requires further attention. This research aimed to explore SU and clinician experiences of SDM in community mental health care.

**Design:**

Qualitative data from a cross‐sectional mixed‐method study is presented. A purposive sample of 18 participants (10 SUs experiencing psychosis, 8 psychiatrists) from one NHS community mental health service, took part in semi‐structured interviews which underwent reflexive thematic analysis.

**Results:**

Participants acknowledged SDM as integral to consultations, with overlaps and disparities in SU and clinician perceptions of the reality in practice. Three themes are reported: (1) knowledge and capacity for SDM, (2) relational dynamics; power and communication in SDM and (3) evaluating the realities of SDM in practice. SUs reported they needed to be in the right frame of mind while clinicians acknowledged they were struggling and it wasn't easy to take medication. SUs also wanted to be more engaged in decisions about their care, involving others in the process, with further clarification about options, and alluded to power asymmetry. Clinicians and SUs stressed that structural pressures (concerning time and resources) limited explanation of treatment options and clinicians reflected on stigma they faced. They questioned if SUs experienced the service they thought they were providing.

**Discussion:**

SU and clinician perceptions often overlapped but at times misaligned, suggesting inconsistency in practicing SDM. Implications for practitioners and policy makers include revisiting how SDM is used in practice, with adequate structural and resource provision, potentially involving others to bridge the gap between experiential and medical knowledge. Limitations are reflected on, however, this research emphasises the importance of consistent use of SDM in community mental health.

**Patient or Public Contribution:**

Service users, academics, and clinicians from the Centre for Public Engagement, Kingston University participated in the research design, development of study materials and data analyses. The study would not be possible without this involvement.

## 1 Introduction

For adults experiencing psychosis, continuous antipsychotic medication remains a preferential treatment for supporting recovery and reducing relapse risk in community mental health [1, 2]. However, in practice, prescribing patterns and adherence are inconsistent, reflecting a complex set of clinical, relational, organisational, and contextual factors, including uneven use of shared decision‐making (SDM) [3, 4, 5, 6, 7]. Recent realist evidence suggests that SDM in psychosis is shaped by medical expertise, perceptions of risk and responsibility, consultation burden, and therapeutic relationships, indicating that implementation is embedded in service context as much as individual practice [3, 6, 8].

SDM is a ‘joint process in which a healthcare professional works together with a person to reach a decision about care, based both on evidence and on the person's individual preferences, beliefs and values’. Although psychiatric decision‐making has historically been clinician‐led, and paternalistic [4] there is now strong policy support for SDM in mental health care [9, 10]. More inclusive and empowering practices are empirically supported, allowing individuals with lived experience to evaluate and voice their preferences and meaningfully engage in decisions regarding their care [11, 12, 13]. However, SDM is not a one‐size‐fits‐all approach and evidence on routine use in community mental health care remains limited, particularly given macro‐level changes to resourcing constraints in England NHS services. SDM is not always the gold‐standard approach or universally desired. Some service users (SUs) view treatment decisions as part of the doctor's domain [14], whereas others express a clear preference for being involved [15, 16] irrespective of their mental health [17] drawing on their experiential understanding of how medication impacts them. Recent reviews demonstrate barriers operating across micro, meso, and macro levels, including communication style, service culture, risk management, and resource constraints [5, 8]. To understand SDM in practice, qualitative research should examine both SU and clinician perspectives.

Communication and relationship quality are central to SDM [18]. Clinician‐reported barriers include jargon‐heavy communication styles with ‘psychiatrist talk’ [19, 20, 21], while collaborative approaches, and accessible follow‐up can support involvement over time [10, 22, 23, 24]. NICE clinical guidance (2021) explicitly recommends that SDM be embedded through training, use of decision aids [25, 26], supporting those who may not be able to engage at a fixed time point.

SU barriers include fluctuating capacity, acute symptoms, and fears of coercion [27, 28]. Findings from SU interviews [7] and clinician interviews [20] indicate how symptom severity and lack of understanding can hinder SU involvement. Understanding SU experiences of their symptoms and decision‐making capabilities and how information is presented is crucial [12]. Strong therapeutic relationships can support trust, reduce disempowerment, and create space for recovery‐oriented discussions and decisions [7, 27, 28].

Practical benefits of SDM are nuanced and require further examination. James and Quirk [29] identified core themes around empowerment, trust building and protecting against coercion. SDM also evidences strong links to increased adherence [30]. Furthermore, [31] described reduction in error rates, help‐seeking behaviours, and stigma resulting from SDM. Nevertheless, despite clinical guidance, policy imperatives, and potential benefits, SDM is still not fully actualised in UK community mental health services [25, 31] and SU and clinician perspectives of its implementation in practice are lacking. To address this evidence gap, in‐depth qualitative research is required.

The aim of this research was to explore perceptions of SDM from SUs experiencing psychosis and clinicians working in UK NHS community mental health.

## 2 Method

### 2.1 Design

The data presented form part of a wider mixed‐method study exploring SU and clinician perceptions of therapeutic relationships, SDM, and medication adherence. The study was developed by the lead researcher (KJ) and supervisory team (MC and IG), in response to an identified gap in the literature on SDM in England NHS community mental health services and KJ's informal conversations with SUs about their involvement in medication decisions. Consistent with the epistemological principles outlined by Lincoln, Lynham, and Guba [32], the study adopted an interpretive approach that prioritised participants' lived realities. Semi‐structured qualitative interviews allowed open‐ended questions and supported the co‐construction of shared meaning. Ontologically, SDM was understood as socially and contextually shaped by SUs' and clinicians' experiences within specific political and clinical settings, including NHS pressures and length of service involvement. Reflexive thematic analysis [33, 34] was used to explore in‐depth narratives about SDM in a single NHS community mental health service in London, England.

### 2.2 Participants

Participants were purposively recruited from those who expressed interest in the quantitative study and were able to discuss their experiences of SDM. The final sample comprised 18 participants (*N* = 10 SUs, *N* = 8 clinicians). Sample size was guided by the analytic approach [33, 34] and principles of information power [35]. Recruitment ceased when sufficient high‐quality information was obtained about SDM in practice, as judged during transcription, initial coding, and collaborative discussions in the supervisory team.

To be included, SUs must have been:

Prescribed antipsychotic medication

Under the care of a NHS community mental health service

Aged 18+

English speaking

Able to provide informed consent (confirmed by referring clinician and KJ)

Clinician inclusion criteria were that they had:

Self‐report of experience prescribing antipsychotic medication

A SU caseload in community mental health services

Tables 1 and 2 present SU and clinician characteristics respectively, which contextualise findings.

**Table 1:** Service user characteristics.

Pseudonym	Gender	Age	Ethnic background	Antipsychotic type	Length of time on current antipsychotic	Length of time with services (years)
Holly	Female	38	White (British)	LAI	2 months	2
Chloe	Female	38	White (British)	Oral	6 years	20
Omar	Male	47	Black (African Caribbean)	LAI	18 months	7
Jamal	Male	65	Middle Eastern	LAI	1 month	8
Anita	Female	21	Asian (Indian)	Oral	14 months	1
Bal	Male	29	Asian (Indian)	Oral	3 months	1
Michael	Male	56	White (Britsh)	Oral	11 years	30+
Rebecca	Female	48	White (British)	LAI	5 years	5
Angelo	Male	38	Black (African Caribbean)	Oral	8 years	8
Victoria	Female	47	White (British)	Oral	15 years	18

Abbreviation: LAI, long‐acting injection.

**Table 2:** Clinician characteristics.

Pseudonym	Gender	Age range	Ethnic background	Occupation	Length of experience (years)
Karina	Female	31–40	White (British)	Trainee	1–5
Eloise	Female	31–40	White (British)	Specialist registrar	11–20
Hussein	Male	31–40	Asian (Other)	Consultant psychiatrist	5–10
Sabine	Female	61+	White (European)	Consultant psychiatrist	21+
Johan	Male	41–50	White (European)	Consultant psychiatrist	11–20
Ada	Female	51–60	Asian (Indian)	Consultant psychiatrist	21+
Gonçalo	Male	41–50	White (European)	Consultant psychiatrist	11–20
David	Male	31–40	White (British)	Trainee	1–5

### 2.3 Data Collection and Ethical Considerations

SUs were identified and referred by clinicians, who assessed both their clinical capacity to engage with research and current involvement in community mental health care. An advert was also displayed in clinic waiting rooms, although no participants were recruited through this route. To reduce any perceived pressure to participate, the researcher (KJ) explained that this study was separate to service provision, and that participants could speak openly about their experiences. SUs were approached in person after a clinic appointment with consent. They were given the participant information sheet (PIS) and offered time to ask questions before providing written informed consent. Approximately 50% of SUs approached by clinicians and the lead researcher consented to participate. Clinicians were introduced to the study during team meetings; and those interested were emailed with the PIS, and consent form before interview.

Interviews were conducted by KJ face‐to‐face in NHS clinic rooms to maximise familiarity and minimise travel burden. SUs were offered a £10 supermarket voucher (through an internal PhD grant) to thank them for their time. There were no minimum requirements for participant remuneration (which is different to involvement and engagement) at the time of data collection. Clinicians were not reimbursed.

Semi‐structured interviews lasted 35–59 min (mean: 42 min) and were audio‐recorded with consent. Interview schedules were informed by the literature and patient and public involvement and engagement (PPIE). Questions explored treatment consultations, experiences of being prescribed antipsychotic medication, information about options received or provided, involvement of other stakeholders, and involvement in final treatment decisions. Clinicians were asked, ‘explain the process of prescribing medication for SUs’ and SUs were asked, ‘explain the process about the information you receive about medication’. Participants were informed they could withdraw without providing reason. A debrief sheet signposted further support, although this was not required. Ethical approval was obtained from South Yorkshire and Humber research ethics committee NHS research ethics committee [ref: 16/YH/0141]. Data management and storage adhered to General Data Protection Regulation (GDPR) requirements.

### 2.4 Reflexivity and Positionality

The researcher (KJ) had previous experience of working in clinical mental health research and interviewing participants with mental health difficulties, but was not a clinician. Rigour was reinforced through PPIE in the research design, and by acknowledging how KJ's position as a young, working‐class woman of colour without lived experience of mental health could shape the research process. For example, KJ held preconceptions of there being a dichotomy between SU and clinician experiences. Attention was paid to avoiding leading questions and remaining open to participants' accounts, while recognising that objectivity was not the aim. The researcher's positionality is further considered below.

### 2.5 Advisory Group and Patient and Public Involvement and Engagement

An advisory group from the Centre for Public Engagement, Kingston University, comprising one SU with lived experience of psychosis, one academic and two clinicians outside of the supervisory team co‐designed the study aims and developed materials (to ensure that the language was clear, accessible and appropriate). PPIE contributors were remunerated. A ‘you said, we did’ approach shaped engagement through two online meetings and back‐and‐forth review and feedback of materials. For example, version one of the research advert was very text‐heavy and not sufficiently accessible, so it was revised to include more images and bullet points. Preliminary research findings were presented at the Centre for Public Engagement research ‘surgery’ where feedback supported sense‐checking of the analysis, providing KJ with opportunity for critical reflections on how they arrived at the final themes.

### 2.6 Data Analysis

Interview data were managed in NVivo 15 and transcribed verbatim. Data were first coded and analysed separately for SUs and clinicians before being brought together in a converged narrative, using reflexive thematic analysis [34]. The approach was inductive, prioritising subjective experiences of SDM over theory‐driven coding. Transcripts were read repeatedly to identify initial patterns, after which codes were generated, from participants' retrospective accounts of SDM. As an outsider with no lived experience of mental health or being a clinician, KJreflected on participants' experiences through phenomenology and narrative inquiry of their journey. Special care was taken to not look for themes in the transcripts that aligned with KJ preconceptions of SDM not being practiced (which had been heard anecdotally during informal conversations prior to the study). Themes and subthemes were developed iteratively from the data, with subthemes presented in vivo to preserve authenticity. Four themes became two then three to better reflect convergence and divergence across SU and clinician accounts. Feedback was sought on theme development with the advisory group and supervisory team, with supervisors as co‐contributing authors.

## 3 Results

Three themes and eight subthemes (presented in Table 3) highlight how experiential and medical knowledge shaped understanding of SDM from SU and clinician perspectives. Exemplar quotes accompany narratives further below, with the participants' pseudonym, gender, and age, and type of antipsychotic and length of time on it (for SUs) or role and length of experience (for clinicians).

**Table 3:** Themes and subthemes.

Themes	Subthemes
Theme one: Knowledge and capacity for SDM	1.1 ‘You got to be in the right frame of mind to read it’ 1.2 ‘They're struggling, (…) sometimes it's very difficult to take medication’ 1.3 ‘There's no great perfect antipsychotic’
Theme two: Relational dynamics: power and communication	2.1 ‘Some sort of power asymmetry’ 2.2 ‘I'd appreciate clarification’ 2.3 ‘Open dialogue’
Theme three: Realities of SDM in practice	3.1 ‘Are patients receiving the service we think we're providing?’ 3.2 ‘Stigma goes both ways’

### 3.1 Theme One: Knowledge and Capacity for SDM

This theme focuses on how knowledge and capability shape engagement in SDM. SUs mobilised experiential knowledge about medication, to make sense of SDM in relation to their recovery, and found they were hindered by fluctuations in capacity. Clinicians through their medical knowledge and expertise, acknowledged the difficulties in SU capacity, medication uptake, and imperfections of antipsychotic medication.

#### 3.1.1 ‘You Got to Be in the Right Frame of Mind to Read It’

Cognitive and emotional states during episodes of illness affected how SUs accessed and understood information and their capacity to engage in SDM;

I was given a lot of information (…) it's quite hard to read it all you know, **you got to be in the right frame of mind to read it**.

(Omar, M, 47, LAI, 7 y)

The psychiatrist did offer to give me a piece of paper with the things written on it (…) I can only read small pieces of text at a time.

(Holly, F, 38, LAI, 2 y)

Omar and Holly were receiving long‐acting injection antipsychotics, which required more tailored, ongoing information. Some SUs also struggled with clinician language, Angelo (M, 38, Oral, 8 y) reporting, *‘sometimes they have their doctor jargon, so it might hard to understand what they're saying’*. Referring to ‘jargon’ indicated need for simpler and accessible communication about medication options. Some relied on relatives to interpret materials, *‘before I started the first medication, my doctor gave me the leaflet and said read up about it, so my son done that’* (Rebecca, F, 48, LAI, 5 y), highlighting the role of informal support networks and potential triadic decision‐making. Given the researcher's prior informal conversations with SUs, it was anticipated that information leaflets would be experienced mainly as inaccessible; however, SUs' accounts showed a more nuanced picture in which comprehension depended on illness phase, format, and support from others.

As symptoms improved, SUs reported feeling clearer and appreciative of the help, indicating the temporal and episodic nature of barriers;

the things that they did to help me get better and back on my feet, now I can see that they were just trying to help.

(Angelo, M, 38, Oral, 8 y)

#### 3.1.2 ‘They're Struggling, (…) Sometimes It's Very Difficult to Take Medication’

Most clinicians recognised fluctuating capacity impacting SUs readiness to engage in SDM and thus sometimes necessitated clinician‐led decisions, *‘patients are said to have less insight into their illness and therefore can't always make the right decision’* (Johan, M, 41–50, CP, 11–20 y). Complexities of SU circumstances were acknowledged *‘…due to the lack of insight, or simply because people have a busy life, **they're struggling,** they've got other stuff going on, and **sometimes it's very difficult to take medication’*** (Gonçalo, M, 41–50, CP, 11–20 y) suggesting sympathy and recognition of personal circumstances and tailored approaches.

Some clinicians acknowledged the expertise and agency of SUs;

…a lot of our patients are experts on their conditions, a lot of them have taken multiple drugs and it's up to both them and us to keep a track of that.

(Karina, F, 31–40, Trainee, 1‐5 y)

…together focus on a biographical approach and try to explore the person's life, if the person works and how does it influence the current situation directly, can be quite useful.

(Hussein, M, 31–40, CP, 5–10 y)

These findings reflect clinicians integrating some experiential (cognitive abilities) and medical (collaborative approaches) knowledge using person‐centred approaches, while facing practical barriers.

#### 3.1.3 ‘There's No Great Perfect Antipsychotic’

Clinicians described antipsychotic prescribing as an uncertain, *‘**there's no great perfect antipsychotic** medication that's kind of side effect free,’* (Eloise, F, 31–40, SpR, 11–20 y) and iterative, *‘sometimes just a case of trial and error and as sad as that is when its more error it can't be avoided sometimes’* (David, M, 31–40, Trainee, 1–5 y).

Some SUs appeared from clinicians' perspectives to defer entirely to them;

at the end of the day most patients go, probably go with a doctor's opinion more than their own, the majority of patients will be probably more swayed by us I think.

(Ada, F, 51–60, CP, 21 y + )

(patients) just want to go ahead with whatever you give them. If the patient then says, ‘I don't care doctor, you're the doctor,’ then I would kind of say, okay this is what I would suggest, this is what is does, these are the side effects what do you think of that. So, I try to work with the patient, as much as I can.

(Gonçalo, M, 41–50, CP, 11–20 y)

Some consultants focused on the importance of follow‐up after initial prescription fitting in with follow‐up models of SDM and using their medical knowledge for aspects of risk management;

we'll review it again (…) called ‘safety netting’ so I tell them, this is what you need to watch out for, this or this happens or if you're worried, or this is not working the way you expect it, get back to us.

(Gonçalo, M, 41–50, CP, 11–20 y)

Most emphasised tailoring decisions around side‐effect profiles, co‐morbidities, and withdrawal goals, suggestion;

you've got somebody who's got a weight problem, then, I don't really want to give them something like Olanzapine which is associated with weight gain.

(Karina, F, 31‐40, Trainee, 1–5 y)

we can support people who want to come off medication by putting reduction strategies in place, rather than just completely withdrawing from medication.

(Johan, M, 41–50, CP, 11‐20 y)

In a similar person‐centred narrative, some clinicians accommodated day‐to‐day SU experiences and complex lives;

we've got people who work full‐time, who achieve incredible things with mental health difficulties, and need to be able to live day to day without side effects and it's a very different place, so I think it is very important that people are involved.

(Karina, F, 31–40, Trainee, 1–5 y)

Clinicians pointed towards other psychological, non‐pharmacological options to support long‐term recovery;

I believe that we have several options in psychiatry to help people (…) I try and make it so that medicating someone is not always the first and only option, I mean in many cases it might be but it's not the be all end all to treatment.

(Johan, M, 41–50, CP, 11–20 y)

These narratives demonstrate clinicians balancing their medical responsibilities, individual preference, and clinical uncertainty, despite the structural pressures and SU specific barriers faced.

### 3.2 Theme Two: Relational Dynamics: Power and Communication

This theme focuses on interpersonal dynamics and how information about medication was provided through the vehicle of a therapeutic relationship. This groups potential operation of power and relational imbalance with communication needs, highlighting barriers to open dialogue.

#### 3.2.1 ‘Some Sort of Power Asymmetry’

Some SUs identified power imbalances that left them feeling excluded from decisions and limited their agency,

With this psychiatrist, I don't feel included in the relationship. I feel remote. I feel on the periphery.

(Michael, M, 56, Oral, 30 y)

the doctor had the power to do the assessment, and knew it was the right medication and all around you need to know yourself if it the right medication for you.

(Bal, M, 29, Oral, 1 y)

These challenged initial assumptions that clinician‐led decision‐making was always experienced as disempowering.

A minority of clinicians described more paternalistic approaches, *‘the final decision would be mine (…) and most of them are agreeable to whatever I say’* (Sabine, F, 61 + , CP, 21 y + ), where Sabine's experience could reflect older consultant‐led‐era perspectives.

Some SUs reflected on the hierarchical nature of multidisciplinary teams, *‘…she's [the psychiatrist] the only one with enough power to make them (…) the other staff I see here more often, who actually know me a bit better.’* (Victoria, F, 37, Oral, 18 y). Michael suggested what could restore inclusion and autonomy;

I'd talk to him in layman's terms, how it impacts upon the client, especially medication, and also I'd try to be less archaic about the relationship (…) I don't want to be his friend. But, I wish he'd include me somewhat.

(Michael, M, 56, Oral, 30 y)

Repeated references to ‘power’ indicate that SUs still felt the need to be more actively involved, where Michael also noted some shift towards greater user empowerment;

Now there's much more user empowerment I find. In the old days **there was some sort of power asymmetry**. The consultant psychiatrist was the man to dispense wisdom. The air about the service was, you listen to him with a bowed head you know? And you couldn't challenge him. But now you can.

(Michael, M, 56, Oral, 30 y)

Given his long‐standing contact with services, his comments may reflect older practices and not recent developments of SDM, but the persistence of power asymmetry narratives suggests that paternalistic approaches may still underpin some consultations.

#### 3.2.2 ‘I'd Appreciate Clarification’

Most SUs expressed strong unmet needs for clear, personalised information about side effects, long‐term implications, and seeking further clarity. Many recognised side effects, *‘all medication have side effects. Some good, some bad but if you want help you just have to put them to the side and take it’* (Rebecca, F, 48, LAI, 5 y) suggesting levels of tolerability. SUs alluded to cost‐benefit analyses, *‘the benefits of the medication outweigh the side effects, it's more helpful’* (Bal, M, 29, Oral, 1 y). However, some felt underinformed of these side effects:

I definitely would've liked to know that I would feel tired all the time (laughs) (…) I don't think anyone told me that this was going to be an issue when I started to take it.

(Angelo, M, 38, Oral, 8 y)

I wouldn't say really had any knowledge about them.

(Anita, F, 21, Oral, 1 y)

Most wanted more clarity on the long‐term impacts, ***…I'd appreciate clarification** (…) All I want to know is, how this medication impacts upon me’* (Michael, M, 56, Oral, 30 y).

#### 3.2.3 ‘Open Dialogue’

Within the overarching theme, the researcher's attention was initially drawn to explicit references to power asymmetry, partially due to prior understanding of psychiatric consultations as hierarchical, yet they were struck by how often clinicians referred to efforts to soften this hierarchy through, clarity, follow‐up, and collaborative language. In contrast to previous subthemes, this subtheme demonstrates how many clinicians emphasised importance of communication with SUs concerning side effects, so they could support as appropriate,

try and encourage patients to be honest about their views on their medication. If you have someone who isn't happy with their medication, we should encourage them to tell us so that we can look at making changes.

(Johan, M, 41‐50, CP, 11‐20 y)

Rooting consultations in bidirectional communication was viewed as necessary,

the open dialogue^1^ initiatives, can be quite useful.

(Hussein, M, 31–40, CP, 5–10)

If we have that open dialogue about what is working for them and what isn't working, we can make the right changes and reductions that help.

(Johan, M, 41–50, CP, 11–20 y)

### 3.3 Theme Three: The Realities of SDM in Practice

Clinicians conceptualised SDM through their medical knowledge, balancing responsibility to empower SUs and make decisions in their best interests, while managing personal stigmas, and NHS structural pressures.

#### 3.3.1 ‘Are Patients Receiving the Service We Think We're Providing?’

Clinicians referred to systemic constraints, such as limited time, staffing, and heavy caseloads;

I don't have the time to explain the medication in detail.

(Gonçalo, M, 41–50, CP, 11–20 y)

one of my most busiest times (…) everyone in the team seems to be stretched quite thin (…) everything is pretty much back‐to‐back here.

(David, M, 31–40, Trainee, 1–5 y)

These reports were consistent across career stages (i.e. trainees and consultant psychiatrists) suggesting shared experiences of strain despite any hierarchical structure which the researcher was particularly drawn to, considering their early‐career stage and own pressures faced. This may have enhanced focus on this during conversations.

SUs similarly considered limited access to clinicians, *‘Its only my doctor I don't get to see regular (…) it's been a long time’* (Omar, M, 47, LAI, 7 y), reflecting wider NHS structural pressures and time constraints, *‘They don't actually have time to sit there and have a full on discussion about how everything works (…) they will just tell you what is good for you to take’* (Victoria, F, 37, Oral, 18 y). This may suggest that passive use of medication was due to limited resources, rather than active shared deliberation.

Some clinicians reflexively considered if their intended practices translated into realities for SUs, *‘is it what we're trying to do here and **are patients receiving the service we think we're providing?**’* (Gonçalo, M, 41–50, CP, 11–20 y) highlighting genuine care for the SU experience.

Considering the realities of SDM in practice, it was observed that a few SUs wished to withdraw from medication, requiring structured support with dose reduction, aligning with clinician observations made in subtheme, 'there's no great perfect antipsychotic';

it would be really amazing if you could just have that kind of support to actually come off (…) I don't know if that's really realistic.

(Chloe, F, 38, Oral, 20 y)

I'd like to explore the option of coming off my medication slowly, I mean I wasn't really told that it would be like a lifetime thing.

(Angelo, M, 38, Oral, 8 y)

#### 3.3.2 ‘Stigma Goes Both Ways’

Reflections on their own positionality emerged for clinicians, *‘(you) make the decisions you think are in the best interests of your patients’* (David, M, 31–40, Trainee, 1–5 y). In an unexpected narrative, some reported feeling stigmatised as ‘pill‐pushers’;

I find it very frustrating, I think stigma goes both ways. Clinicians are often stigmatised about being pill‐pushing and actually in my experience most of us aren't, all we're ever trying to do, is use the best of our knowledge and experience of helping people get out of difficult situations (…) we certainly don't want to chemically restrain our patients (…) I guess sometimes it can feel quite, hurtful's the wrong word but, it is tough to be on the receiving end of that.

(Eloise, F, 31–40, SpR, 11–20 y)

These accounts highlight tensions between clinicians delivering person‐centred care and experiences of stigma, which could impact their wellbeing. The finding was unexpected for the researcher, expectations for clinicians to do their job to the best of their ability and most informal conversations centred around individual SDM rather than wider structural pressures.

Overall, the data contributing to this theme was initially approached with an interest in why SDM was not occurring more consistently, the interviews repositioned this less as a matter of individuals not practicing SDM and more as an organisational reality shaped by time, strain, and risk management.

## 4 Discussion

This study explored SU and clinician perceptions of SDM in NHS community mental health care. Reflexive thematic analysis showed that although both groups recognised the value of SDM, their experiences often diverged, shaped by differences in experiential and medical knowledge. SUs emphasised power asymmetry, difficulty accessing information, and feelings of exclusion; while clinicians highlighted communication, person centred approaches, and stigma. Shared recognition of the practical realities of service pressures were evident, suggesting SDM is shaped less by endorsement of its principles than by how knowledge, capacity, and organisational constraints interact in practice.

### 4.1 Accessible Information, Capacity and Autonomy

Accessible, tailored information was central to meaningful SDM, but SUs often found complex materials difficult to engage with during periods of cognitive distress. This aligns with NICE guidance which emphasises provision of accessible, tailored information, with time and support to evaluate it [10]. It also echoes evidence that SUs want clearer, personalised information about medication options [37]. Accounts of SUs wanting more *‘clarification’* about medication or that they were not in the *‘right frame of mind’* to understand it, extends work of Kaminskiy, Ramon, and Morant [38] who reported SUs being unable to understand information during early stages of illness. This reinforces that SDM should not be treated as a one‐off eventand aligns with follow‐up models of SDM [24].

Involvement of others to help make sense of treatment information suggests SDM in psychosis functions as a relational rather than individual process [5, 39, 40]. Efforts for caregiver involvement and triadic decision‐making are increasing in healthcare, supporting this direction in the UK context [41, 42, 43].

Clinicians recognised fluctuating capacity, uncertainty, and the need to revisit decisions after the acute phase, supporting prior work [12, 44]. Rather than viewing reduced capacity as a reason to abandon SDM, these accounts support approaches that treat autonomy as something that can be restored, maintained, and revisited over time through follow‐up, advance directives, and the involvement of others [43, 45, 46, 47]. In developing the theme, the researcher's understanding shifted from a narrow concern with information quality to a broader understanding of timing, illness phase, and support as determinants of use of information.

### 4.2 Power Asymmetry, the Therapeutic Relationship and Clinician Stigma

Power asymmetry was a consistent feature of SU accounts, particularly where consultations were shaped by jargon, hierarchy, and limited space for challenge with many feeling peripheral to decision‐making. Clinicians, by contrast, appeared concerned with whether SUs were receiving the service they thought they were providing, reflecting potential gaps between intent and experience. Further reflection meant this appeared less like a simple communication disparity, but rather a structural scarcity in resources to practice SDM alongside a mismatch in how each group understood authority, trust, and involvement [6].

Mechanisms of power are well recognised in psychiatric decision‐making, but power remains a complex phenomenon where potential uses and abuses are not fully understood, requiring further study [31, 48, 49, 50]. The current findings support the view that SDM requires an ongoing balance of empowerment and responsibility, built on trust and relational continuity [51]. However, the factors that support this relationship in community mental health settings remain underexplored.

Clinicians expressed emotional strain, and feeling stigmatised as ‘pill‐pushers’. Although a view less commonly documented, it aligns with literature on declining morale, risk assessment, workload pressure, and burnout in mental health services [52, 53, 54]. Feeling stigmatised for trying to support recovery may influence how clinicians communicate and how willing they are to engage in open discussion about treatment options. This may suggest perceived stigma and clinician well‐being are not separate from SDM but part of the condition that makes it possible. An initial simple reading during the analysis of exclusion as a SU contention shifted to a shared but unevenly experienced system of authority, risk, and strained professional identity.

### 4.3 Service Pressures and Uncertainty

Systemic constraints and organisational realities were evident. Both SUs and clinicians described limited consultation time, heavy workload, and the pressures of working within stretched NHS services, mirroring wider concerns about gaps in community mental health provision [55, 56, 57]. Some clinicians still reported adopting open dialogue approaches with the time they had [36]. Although time is often cited as a barrier to SDM, evidence suggests that it only minimally increases consultation length [26], with practical tools such as decision aids and assessment tools such as the Antipsychotic Medication Management Fidelity scale [58] helping support implementation. There is increasing recognition of the value of decision aids in mental health care, including process models of SDM that treat decision‐making as ongoing rather than confined to a single consultation [10, 23, 24, 26, 59, 60, 61]. Peer support may offer efficiency to support SUs involvement in their treatment [40, 62].

Clinicians also described antipsychotic prescribing as inherently uncertain; an iterative process relying on trial and error. This uncertainty matters because it makes transparent discussion about medication changes, tapering, and withdrawal important. However, some SUs reported they would have liked but did not receive support for this, reinforcing the need for clearer guidance on withdrawal and long‐term treatment effects [63, 64]. Both SUs and clinicians acknowledged the wider view that long‐term medication is not the be all end all to treatment [65, 66, 67]. Together, the findings suggest that SDM should be understood as an iterative process requiring time, follow‐up, and realistic support for practice in community mental health.

### 4.4 Strengths and Limitations

The study's strengths lie in its dual‐perspective design, PPIE involvement, and reflexive approach, enabling a nuanced exploration of how experiential and medical knowledge intersect, diverge, and shape decision‐making. Although previous research has shown that SDM can be shaped by power, communication, and uncertainty [68], this study adds to the literature by showing how these issues are experienced and interpreted by SUs and clinicians in England NHS community mental health services and can be further explored with guideline changes [10]. In particular, the study extends existing work by showing that clinician stigma, workload strain, and concerns about risk are not about disempowerment but part of the conditions under which SDM is attempted, providing further support for acknowledging individual and structural factors underpinning SDM.

However, the absence of clinician‐SU dyads means that some misalignment may reflect different clinical contexts rather than direct relational dynamics. The study was conducted in a single NHS service and drew on retrospective interviews collected in 2019, before some more recent developments in SDM guidance [9, 10]. SUs were referred by clinicians which may reflect a singular set of experiences of SDM and although there were diverse experiences expressed, this could limit representation of SDM in practice. Although generalisability is not the goal, some of the findings should be interpreted in relation to local and temporal context. Nonetheless, the research offers rich insight into the realities of practicing SDM in a comparative approach.

### 4.5 Implications and Future Direction

Future research should explore clinician‐SU dyads and use longitudinal designs, from initial consultation to follow‐up, to better understand how decision‐making capacity, relational power, and recovery change over time. Further work is also needed on clinician stigma, wellbeing, and the organisational conditions that support or inhibit SDM. At the UK policy level, clinicians require adequate time, staffing, and workload support to enable SDM [6], alongside accessible, co‐produced decision aids for antipsychotic prescribing, withdrawal, and crisis planning [10, 61, 62]. National prescribing guidance should also be updated to reflect the growing evidence on tapering and withdrawal [67], while routine use of process models and advance directives could help preserve autonomy during episodes of reduced capacity [60].

In practice, clinicians should be supported to revisit information and follow‐up decisions at multiple points, concerning discussions about medication options, side effect risks and withdrawal, given SUs may not be in the ‘right frame of mind’ during acute illness. This should be underpinned by relational practices that aim to reduce power asymmetry, creating a culture where SUs feel more empowered to question, challenge, or express preferences. By prioritising accessible, ‘jargon‐free’ communications, open‐dialogue, and the involvement of others where appropriate including triadic decision‐making, these changes can enhance autonomy, empower SUs, and improve adherence.

## 5 Conclusion

This study explored SU and clinician experiences of SDM within a NHS community mental health service. Although both groups valued SDM, their accounts differed due to experiential and medical knowledge underpinned by acute illness, communication, involvement of others, and service pressures.

SUs highlighted the need for accessible, personalised information and repeated discussion over time, considering long‐term impact of medication and acknowledging how their cognitive and emotional difficulties could make medication information hard to understand. Frequent reports of power asymmetry, jargon, exclusion, and hierarchical consultation structures, suggest that SDM needs to be more explicitly relational, inclusive and restorative of autonomy including supporting triadic decision‐making where appropriate.

Both SUs and clinicians framed SDM in relation to fluctuating capacity and NHS pressures, including heavy caseloads, limited consultation time, and the uncertainties of antipsychotic prescribing and reduction. Clinicians described the emotional burden and stigma of being perceived as pill‐pushers, indicating their wellbeing may be important for sustaining person‐centred care.

Overall, the findings suggest that SDM in community mental health cannot be viewed at the individual level but any inconsistency in practice is context‐dependent on wider systematic pressures. Improving accessible information, decision aids, follow‐up, and triadic approaches may bridge experiential and medical knowledge to empower SUs to engage in decisions, reduce clinician stigma, and minimise power asymmetry for better implementation of SDM and supporting SU recovery.

## Author Contributions

**Karishma Jivraj:** conceptualisation, investigation, funding acquisition, writing – original draft, methodology, writing – review and editing, formal analysis, project administration, data curation. **Iris Gault:** conceptualisation, supervision, writing – review and editing, supervision. **Mary Chambers:** conceptualisation, writing – review and editing, supervision.

## Ethics Statement

This study received ethical approval from Kingston Research Ethics Committee (Sponsor Reference: 16.0037) and NHS South Yorkshire and Humber research ethics committee (16/YH/0141).

## Conflicts of Interest

The authors declare no conflicts of interest.

## Data Availability

Data can be made available by authors upon reasonable request.

## Footnotes

1 Open Dialogue approach [31], developed in Finland, is a model of mental health care delivery involving a consistent SU, family and social network approach in the treatment of the SU.

## References

[1] National Institute for Health and Care Excellence (NICE). (2014). Psychosis and schizophrenia in adults: Treatment and management (Clinical guideline CG178). 31869041

[2] NHS.UK (n.d.) . Psychosis: Overview. accessed, at https://www.nhs.uk/mental-health/conditions/psychosis/overview/.

[3] Y. Aoki , L. Perestelo‐Pérez , H. Matsuura , and Y. Zisman‐Ilani , “Shared Decision‐making In Mental Healthcare.” Oxford University Press (Oxford textbook of shared decision‐making in healthcare, 2025), 143.

[4] C. Charles , A. Gafni , and T. Whelan , “Shared Decision‐Making in the Medical Encounter: What Does It Mean? (Or It Takes at Least Two to Tango),” Social Science & Medicine (1982) 44, no. 5 (1997): 681–692, 10.1016/S0277-9536(96)00221-3. 9032835

[5] M. Chmielowska , Y. Zisman‐Ilani , R. Saunders , and S. Pilling , “Trends, Challenges, and Priorities for Shared Decision Making in Mental Health: The First Umbrella Review,” International Journal of Social Psychiatry 69, no. 4 (2023): 823–840, 10.1177/00207640221140291. 36680367 PMC10240653

[6] P. W. Corrigan and V. Kharbanda , “Practical guide to shared decision making for mental health,” in Shared Decision Making and Serious Mental Illness, eds. In. P. W. Corrigan . American Psychological Association, 2026), 153–192. 10.1037/0000478-006.

[7] E. Kaminskiy , Y. Zisman‐Ilani , N. Morant , and S. Ramon , “Barriers and Enablers to Shared Decision Making in Psychiatric Medication Management: A Qualitative Investigation of Clinician and Service Users' Views,” Frontiers in Psychiatry 12 (2021): 678005, 10.3389/fpsyt.2021.678005. 34220584 PMC8245843

[8] I. Fitzgerald , J. Howe , I. Maidment , et al., “From Idealist to Realist—Designing and Implementing Shared Decision‐Making Interventions in the Choice of Antipsychotic Prescription in People Living With Psychosis (Shape): A Realist Review (Part 2—Designing Sdm Interventions: Optimizing Design and Local Implementation),” Schizophrenia Bulletin 51, no. 4 (2025): 932–948, 10.1093/schbul/sbaf059. 40396338 PMC12236318

[9] NHS England . (2019). Shared decision‐making: Summary guide. accessed, at https://www.england.nhs.uk/wp-content/uploads/2019/01/shared-decision-making-summary-guide-v1.pdf.

[10] National Institute for Health and Care Excellence (NICE) . (2021). Shared decision‐making (Guideline NG197). 34339147

[11] A. Coulter and A. Collins . Making Shared Decision‐making a Reality: No Decision About Me, Without Me. The King's Fund (2011).

[12] E. W. Haugom , B. Stensrud , G. Beston , T. Ruud , and A. S. Landheim , “Mental Health Professionals' Experiences With Shared Decision‐Making for Patients With Psychotic Disorders: A Qualitative Study,” BMC Health Services Research 20 (2020): 1093, 10.1186/s12913-020-05949-1. 33246451 PMC7694931

[13] E. C. Thomas , S. Ben‐David , E. Treichler , et al., “A Systematic Review of Shared Decision‐Making Interventions for Service Users With Serious Mental Illnesses: State of the Science and Future Directions,” Psychiatric Services 72, no. 11 (2021): 1288–1300, 10.1176/appi.ps.202000429. 34369801 PMC8570969

[14] D. C. Stewart , G. B. Anthony , and R. Chesson , “It's Not My Job. I'm the Patient Not the Doctor’: Patient Perspectives on Medicines Management in the Treatment of Schizophrenia,” Patient Education and Counseling 78, no. 2 (2010): 212–217, 10.1016/j.pec.2009.06.016. 19665339

[15] S. Farrelly , G. Brown , D. Rose , et al., “What Service Users With Psychotic Disorders Want in a Mental Health Crisis or Relapse: Thematic Analysis of Joint Crisis Plans,” Social Psychiatry and Psychiatric Epidemiology 49, no. 10 (2014): 1609–1617, 10.1007/s00127-014-0869-1. 24691492 PMC4165869

[16] N. Morant , K. Azam , S. Johnson , and J. Moncrieff , “The Least Worst Option: User Experiences of Antipsychotic Medication and Lack of Involvement in Medication Decisions in a Uk Community Sample,” Journal of Mental Health 27, no. 4 (2018): 322–328, 10.1080/09638237.2017.1370637. 28857636

[17] J. Hamann , F. Holzhüter , S. Blakaj , et al., “Implementing Shared Decision‐Making on Acute Psychiatric Wards: A Cluster‐Randomized Trial With Inpatients Suffering From Schizophrenia (Sdm‐Plus),” Epidemiology and Psychiatric Sciences 29 (2020): e137, 10.1017/S2045796020000505. 32539907 PMC7303792

[18] S. Becher , F. Holzhüter , S. Heres , and J. Hamann , “Barriers and Facilitators of Shared Decision Making in Acutely Ill Inpatients With Schizophrenia,” Health Expectations 24, no. 5 (2021): 1737–1746, 10.1111/hex.13313. 34258833 PMC8483208

[19] R. E. Drake , D. Cimpean , and W. C. Torrey , “Shared Decision Making in Mental Health: Prospects for Personalized Medicine,” Dialogues in Clinical Neuroscience 11, no. 4 (2009): 455–463, 10.31887/DCNS.2009.11.4/redrake. 20135903 PMC3181931

[20] C. Y. Lin , L. Renwick , and K. Lovell , “Health Professionals' Perspectives on Shared Decision‐Making in Secondary Mental Healthcare: A Qualitative Study,” Journal of Mental Health 31, no. 5 (2022): 709–715, 10.1080/09638237.2021.2022608. 34978256

[21] R. McCabe , H. Khanom , P. Bailey , and S. Priebe , “Shared Decision‐Making in Ongoing Outpatient Psychiatric Treatment,” Patient Education and Counseling 91, no. 3 (2013): 326–328, 10.1016/j.pec.2012.12.020. 23414657

[22] G. Elwyn , M. A. Durand , J. Song , et al., “A Three‐Talk Model for Shared Decision‐Making: Multistage Consultation Process,” BMJ 359 (2017): j4891, 10.1136/bmj.j4891. 29109079 PMC5683042

[23] G. Elwyn , D. Frosch , R. Thomson , et al., “Shared Decision Making: A Model for Clinical Practice,” Journal of General Internal Medicine 27, no. 10 (2012): 1361–1367, 10.1007/s11606-012-2077-6. 22618581 PMC3445676

[24] K. Grim , D. Rosenberg , P. Svedberg , and U. K. Schön , “Shared Decision‐Making in Mental Health Care‐A User Perspective on Decisional Needs in Community‐Based Services,” International Journal of Qualitative Studies on Health and Well‐Being 11 (2016): 30563, 10.3402/qhw.v11.30563. 27167556 PMC4862955

[25] S. Ramon and E. Y. W. Yeung , “United Kingdom: The Place of Shared Decision‐Making.” Research Handbook on Mental Health Policy (Edward Elgar Publishing, 2022), 258–271.

[26] D. Stacey , F. Légaré , K. Lewis , et al., “Decision Aids for People Facing Health Treatment or Screening Decisions,” Cochrane Database of Systematic Reviews 2017, no. 4 (2017): CD001431, 10.1002/14651858.CD001431.pub5. PMC6478132 28402085

[27] L. Beames and J. Onwumere , “Risk Factors Associated With Use of Coercive Practices in Adult Mental Health Inpatients: A Systematic Review,” Journal of Psychiatric and Mental Health Nursing 29, no. 2 (2022): 220–239, 10.1111/jpm.12757. 33835622

[28] N. Morant , E. Kaminskiy , and S. Ramon , “Shared Decision Making for Psychiatric Medication Management: Beyond the Micro‐Social,” Health Expectations 19, no. 5 (2016): 1002–1014, 10.1111/hex.12392. 26260361 PMC5053275

[29] K. James and A. Quirk , “The Rationale for Shared Decision Making in Mental Health Care: A Systematic Review of Academic Discourse,” Mental Health Review Journal 22, no. 3 (2017): 152–165, 10.1108/MHRJ-01-2017-0009.

[30] L. Burns , A. L. da Silva , and A. John , “Shared Decision‐Making Preferences in Mental Health: Does Age Matter? A Systematic Review,” Journal of Mental Health 30, no. 5 (2021): 634–645, 10.1080/09638237.2020.1793124. 32662713

[31] M. Slade , “Implementing Shared Decision Making in Routine Mental Health Care,” World Psychiatry 16, no. 2 (2017): 146–153. 10.1002/wps.20412. 28498575 PMC5428178

[32] Y. S. Lincoln , S. A. Lynham , and E. G. Guba , “Paradigmatic Controversies, Contradictions, and Emerging Confluences.” in The SAGE Handbook of Qualitative Research, eds. In. N. K. Denzin and Y. S. Lincoln . SAGE, 2011). 4th ed., 97–128).

[33] V. Braun and V. Clarke , “Reflecting on Reflexive Thematic Analysis,” Qualitative Research in Sport, Exercise and Health 11, no. 4 (2019): 589–597, 10.1080/2159676X.2019.1628806.

[34] V. Braun and V. Clarke , Thematic Analysis: A Practical Guide (SAGE Publications, 2021).

[35] K. Malterud , V. D. Siersma , and A. D. Guassora , “Sample Size in Qualitative Interview Studies: Guided by Information Power,” Qualitative Health Research 26, no. 13 (2016): 1753–1760, 10.1177/1049732315617444. 26613970

[36] J. Seikkula , B. Alakare , J. Aaltonen , J. Holma , A. Rasinkangas , and V. Lehtinen , “Open Dialogue Approach: Treatment Principles and Preliminary Results of a Two‐Year Follow‐Up on Fi Rst Episode Schizophrenia,” Ethical Human Sciences and Services 5 (2003): 163–182.

[37] M. Laitila , J. Nummelin , T. Kortteisto , and A. Pitkänen , “Service Users' Views Regarding User Involvement in Mental Health Services: A Qualitative Study,” Archives of Psychiatric Nursing 32, no. 5 (2018): 695–701, 10.1016/j.apnu.2018.03.009. 30201197

[38] E. Kaminskiy , S. Ramon , and N. Morant , “Exploring Shared Decision‐Making in Psychiatric Medication Management,” In S. Walker (Ed.), Modern Mental Health Care: Understanding, Supporting and Transforming Services (2013), 10.4324/9781041056270-5.

[39] S. Gillard , “Peer Support in Mental Health Services: Where Is the Research Taking Us, and Do We Want to Go There?,” Journal of Mental Health 28, no. 4 (2019): 341–344, 10.1080/09638237.2019.1608935. 31070066

[40] Y. Zisman‐Ilani and L. Byrne , “Shared Decision Making and Peer Support: New Directions for Research and Practice,” Psychiatric Services 74, no. 4 (2023): 427–428, 10.1176/appi.ps.20220407. 36164768 PMC10040459

[41] J. Hamann and S. Heres , “Why and How Family Caregivers Should Participate in Shared Decision Making in Mental Health,” Psychiatric Services 70, no. 5 (2019): 418–421, 10.1176/appi.ps.201800362. 30784381

[42] S. Olsena , I. Stars , E. E. Rozenberga , K. Konstantinova , and K. Baidina , “Triadic Perspectives on Decision Making in Psychiatry: A Qualitative Study on Service Users, Caregivers and Healthcare Professionals in Latvia,” Healthcare 13, no. 12 (2025): 1416, 10.3390/healthcare13121416. 40565443 PMC12192966

[43] F. Schuster , F. Holzhüter , S. Heres , and J. Hamann , “Triadic' Shared Decision Making in Mental Health: Experiences and Expectations of Service Users, Caregivers and Clinicians in Germany,” Health Expectations 24, no. 2 (2021): 507–515, 10.1111/hex.13192. 33450125 PMC8077125

[44] J. Hamann , S. Kohl , R. McCabe , et al., “What Can Patients Do to Facilitate Shared Decision Making? A Qualitative Study of Patients With Depression or Schizophrenia and Psychiatrists,” Social Psychiatry and Psychiatric Epidemiology 51, no. 4 (2016): 617–625, 10.1007/s00127-015-1089-z. 26155899

[45] G. Elwyn , “Shared Decision‐Making: What Is the Work?,” Patient Education and Counseling 104, no. 7 (2021): 1591–1595, 10.1016/j.pec.2020.11.032. 33353840

[46] E. Braun , A. S. Gaillard , J. Vollmann , J. Gather , and M. Scholten , “Mental Health Service Users' Perspectives on Psychiatric Advance Directives: A Systematic Review,” Psychiatric Services 74, no. 4 (2023): 381–392, 10.1176/appi.ps.202200003. 36128696

[47] C. M. Wilder , J. W. Swanson , R. J. Bonnie , T. Wanchek , L. McLaughlin , and J. Richardson , “A Survey of Stakeholder Knowledge, Experience, and Opinions of Advance Directives for Mental Health in Virginia,” Administration and Policy in Mental Health and Mental Health Services Research 40, no. 3 (2013): 232–239, 10.1007/s10488-011-0401-9. 22240937

[48] E. R. Carrotte , M. E. Hartup , B. Lee‐Bates , and M. Blanchard , “I Think That Everybody Should Be Involved”: What Informs Experiences of Shared Decision‐Making in Supporting People Living With Schizophrenia Spectrum Disorders?,” Patient Education and Counseling 104, no. 7 (2021): 1583–1590, 10.1016/j.pec.2020.11.012. 33229188

[49] R. Stenhouse and C. Muirhead , “Developing and maintaining therapeutic relationships.” Psychiatric and Mental Health Nursing (Routledge, 2017), 29–38.

[50] E. B. H. Treichler , B. A. Rabin , A. N. Cohen , and G. A. Light , “How Shared Is Shared Decision Making? Reaching the Full Potential of Patient‐Clinician Collaboration in Mental Health,” Harvard Review of Psychiatry 29, no. 5 (2021): 361–369, 10.1097/HRP.0000000000000304. 34352846 PMC12478557

[51] L. S. Beyene , E. Severinsson , B. S. Hansen , and K. Rørtveit , “Shared Decision‐Making—Balancing Between Power and Responsibility as Mental Health‐Care Professionals in a Therapeutic Milieu,” SAGE Open Nursing 4 (2018): 2377960817752159, 10.1177/2377960817752159. 33415187 PMC7774362

[52] K. O'Connor , D. Muller Neff , and S. Pitman , “Burnout in Mental Health Professionals: A Systematic Review and Meta‐Analysis of Prevalence and Determinants,” European Psychiatry 53 (2018): 74–99, 10.1016/j.eurpsy.2018.06.003. 29957371

[53] S. Wallbank , “The Health and Wellbeing of Nhs Staff in a Community Setting,” British Journal of Healthcare Management 31, no. 2 (2025): 1–8, 10.12968/bjhc.2024.0076.

[54] P. T. Yanos , B. Vayshenker , J. S. DeLuca , and L. K. O'Connor , “Development and Validation of a Scale Assessing Mental Health Clinicians' Experiences of Associative Stigma,” Psychiatric Services 68, no. 10 (2017): 1053–1060, 10.1176/appi.ps.201600553. 28617207

[55] E. W. Haugom , B. Stensrud , G. Beston , T. Ruud , and A. S. Landheim , “Experiences of Shared Decision Making Among Patients With Psychotic Disorders in Norway: A Qualitative Study,” BMC Psychiatry 22, no. 1 (2022): 192, 10.1186/s12888-022-03849-8. 35300633 PMC8932170

[56] R. Pedley , C. McWilliams , K. Lovell , et al., “Qualitative Systematic Review of Barriers and Facilitators to Patient‐Involved Antipsychotic Prescribing,” BJPsych Open 4, no. 1 (2018): 5–14, 10.1192/bjo.2017.5. 29388908 PMC6020265

[57] UK Parliament . (2025). Community mental health services: Fourth report of session 2024–2026. accessed, at https://publications.parliament.uk/pa/cm5901/cmselect/cmhealth/1769/report.html.

[58] T. Ruud , K. Drivenes , R. E. Drake , et al., “The Antipsychotic Medication Management Fidelity Scale: Psychometric Properties,” Administration and Policy in Mental Health and Mental Health Services Research 47, no. 6 (2020): 911–919, 10.1007/s10488-020-01018-1. 32030595 PMC7547997

[59] Y. Aoki , T. Tsuboi , Y. Takaesu , et al., “Development and Field Testing of a Decision Aid to Facilitate Shared Decision Making for Adults Newly Diagnosed With Attention‐Deficit Hyperactivity Disorder,” Health Expectations 25, no. 1 (2022): 366–373, 10.1111/hex.13393. 34856044 PMC8849269

[60] C. Gurtner , J. M. G. A. Schols , C. Lohrmann , R. J. G. Halfens , and S. Hahn , “Conceptual Understanding and Applicability of Shared Decision‐Making in Psychiatric Care: An Integrative Review,” Journal of Psychiatric and Mental Health Nursing 28, no. 4 (2021): 531–548, 10.1111/jpm.12712. 33191536

[61] Y. Zisman‐Ilani , M. Parker , E. C. Thomas , et al., “Usability and Feasibility of the Antipsychotic Medication Decision Aid in a Community Program for First‐Episode Psychosis,” Psychiatric Services 75, no. 8 (2024): 807–811, 10.1176/appi.ps.20230230. 38477836

[62] Y. Zisman‐Ilani , D. Shern , P. Deegan , et al., “Continue, Adjust, or Stop Antipsychotic Medication: Developing and User Testing An Encounter Decision Aid for People With First‐Episode and Long‐Term Psychosis,” BMC Psychiatry 18, no. 1 (2018): 142, 10.1186/s12888-018-1707-x. 29788933 PMC5963160

[63] M. A. Horowitz , S. Jauhar , S. Natesan , R. M. Murray , and D. Taylor , “A Method for Tapering Antipsychotic Treatment That May Minimize the Risk of Relapse,” Schizophrenia Bulletin 47, no. 4 (2021): 1116–1129, 10.1093/schbul/sbab017. 33754644 PMC8266572

[64] J. Read , “The Experiences of 585 People When They Tried to Withdraw From Antipsychotic Drugs,” Addictive Behaviors Reports 15 (2022): 100421, 10.1016/j.abrep.2022.100421. 35434245 PMC9006667

[65] B. Keogh , E. Murphy , L. Doyle , G. Sheaf , M. Watts , and A. Higgins , “Mental Health Service Users Experiences of Medication Discontinuation: A Systematic Review of Qualitative Studies,” Journal of Mental Health 31, no. 2 (2022): 227–238, 10.1080/09638237.2021.1922644. 34126035

[66] A. P. Morrison , H. Law , L. Carter , et al., “Antipsychotic Drugs Versus Cognitive Behavioural Therapy Versus a Combination of Both in People With Psychosis: A Randomised Controlled Pilot and Feasibility Study,” Lancet Psychiatry 5, no. 5 (2018): 411–423, 10.1016/S2215-0366(18)30096-8. 29605187 PMC6048761

[67] J. Moncrieff , N. Crellin , J. Stansfeld , et al., “Antipsychotic Dose Reduction and Discontinuation Versus Maintenance Treatment in People With Schizophrenia and Other Recurrent Psychotic Disorders in England (The Radar Trial): An Open, Parallel‐Group, Randomised Controlled Trial,” Lancet Psychiatry 10, no. 11 (2023): 848–859, 10.1016/S2215-0366(23)00258-4. 37778356

[68] L. Mikesell , E. Bromley , A. S. Young , P. Vona , and B. Zima , “Integrating Client and Clinician Perspectives on Psychotropic Medication Decisions: Developing a Communication‐Centered Epistemic Model of Shared Decision Making for Mental Health Contexts,” Health Communication 31, no. 6 (2016): 707–717, 10.1080/10410236.2014.993296. 26529605

